# NLRC4 methylation and its response to intravenous immunoglobulin therapy in Kawasaki disease: a case control study

**DOI:** 10.1186/s12887-024-04672-8

**Published:** 2024-03-16

**Authors:** Beirong Yu, Bangxu Zheng, Yu Shen, Yijing Shen, Haiyan Qiu, Ling Wu, Yuanling Chen, Xiaohong Cai, Junhua Wu, Qingxiao Hong

**Affiliations:** 1 Department of Pediatrics, Ningbo Women and Children’s Hospital, Ningbo, Zhejiang China; 2 Department of Reproductive Medicine, Ningbo Women and Children’s Hospital, Ningbo, Zhejiang China; 3 Department of Scientific Research, Ningbo Women and Children’s Hospital, Ningbo, Zhejiang China; 4 Medical School, Ningbo University, Ningbo, Zhejiang China; 5 Department of psychiatry, Affiliated Kangning Hospital of Ningbo University, Ningbo, 315201 Zhejiang China; 6 Key Laboratory of Addiction Research of Zhejiang Province, Ningbo Kangning Hospital, Ningbo, 315201 Zhejiang China

**Keywords:** Kawasaki disease, NLRC4, Methylation, Intravenous immunoglobulin

## Abstract

**Background:**

Kawasaki disease (KD) is a systemic vasculitis accompanied by many systemic physiological and biochemical changes. Elucidating its molecular mechanisms is crucial for diagnosing and developing effective treatments. NLR Family CARD Domain Containing 4 (NLRC4) encodes the key components of inflammasomes that function as pattern recognition receptors. The purpose of this study was to investigate the potential of NLRC4 methylation as a biomarker for KD.

**Methods:**

In this study, pyrosequencing was utilized to analyze NLRC4 promoter methylation in blood samples from 44 children with initial complete KD and 51 matched healthy controls. Methylation at five CpG sites within the NLRC4 promoter region was evaluated.

**Results:**

Compared to controls, NLRC4 methylation significantly decreased in KD patients (CpG1: *p* = 2.93E-06; CpG2: *p* = 2.35E-05; CpG3: *p* = 6.46E-06; CpG4: *p* = 2.47E-06; CpG5: *p* = 1.26E-05; average methylation: *p* = 5.42E-06). These changes were significantly reversed after intravenous immunoglobulin (IVIG) treatment. ROC curve analysis demonstrated remarkable diagnostic capability of mean NLRC4 gene methylation for KD (areas under ROC curve = 0.844, sensitivity = 0.75, *p* = 9.61E-06, 95% confidence intervals were 0.762–0.926 for mean NLRC4 methylation). In addition, NLRC4 promoter methylation was shown to be significantly negatively correlated with the levels of central granulocyte percentage, age, mean haemoglobin quantity and mean erythrocyte volume. Besides, NLRC4 promoter methylation was positively correlated with lymphocyte percentage, lymphocyte absolute value.

**Conclusions:**

Our work revealed the role of peripheral NLRC4 hypomethylation in KD pathogenesis and IVIG treatment response, could potentially serve as a treatment monitoring biomarker, although its precise functions remain to be elucidated.

**Supplementary Information:**

The online version contains supplementary material available at 10.1186/s12887-024-04672-8.

## Background

Kawasaki disease (KD), also known as mucocutaneou lymph node syndrome, it is an acute fever and rash disease mainly characterized by systemic vascular lesions. KD mainly involves small and medium blood vessels throughout the body, it can lead to coronary artery lesion (CAL), such as coronary artery dilation, which can be further developed into coronary artery aneurysm (CAA) in severe cases, CAA is prone to serious complications such as coronary thrombosis, calcification, stenosis, obstruction, and even tumor rupture [1–3]. The majority of children patients self-resolve in within 1–2 weeks without sequelae. While, 5%, 6% and 2% of KD patients is shown to be complicated by CAA, shock or macrophage activation syndrome (MAS), respectively [4]. The epidemiology of KD varies by geographic region, ethnic and seasonality. In addition, studies have shown that KD has obvious familial aggregation, children of parents with a KD history are twice as likely to develop to KD, and the clinical symptoms are more severe. The risk for the siblings of affected KD children is 10 times higher than that of the general population [5, 6], which showed the genetic characteristic. Currently, intravenous immunoglobulin (IVIG) combined with high-dose aspirin is the standard and best treatment for KD patients. The incidence of coronary complications can be reduced to 3-5% if the patients receive timely administration of IVIG treatment within 10 days of onset of fever [7].

Although a handful of genetic markers have been identified in KD [8–10], the pathogenesis of KD remains unclear. Environmental factors are also shown to be related to KD. Recent developments pointing toward promising therapeutics that target genes with aberrant DNA methylation have been reported, especially genes associated with inflammasome activity might contribute to the risk of KD.

Nucleotide-Binding Oligomerization Domain, Leucine Rich Repeat and CARD Domain Containing 4 (NLRC4) encodes the key components of inflammasome that function as pattern recognition receptors, NLRC4 plays a critical role in innate immune responses to a variety of pathogens, infection, tissue damage, and other cellular stress through multiprotein inflammasome complexes [11]. Gain-of-function mutations in NLRC4 is linked to autoinflammatory disorders in humans. Previous study had reported the up-regulated and hypomethylation in NLRC4 in acute-phase KD [12]. However, no additional studies have examined the possibility of NLRC4 as a molecular marker for KD.

## Methods and materials

### Patients and sample preparation

A total of 44 children with initial complete KD and 51 matched healthy controls were recruited from Ningbo Women and Children’s Hospital. The number of CAL cases was 8 (18.2%) in the KD group and none in the control group.

Blood samples of 15 patients that with significant improvement after IVIG treatment were collected for pre- and post-medication analysis. KD cases were diagnosed by experienced pediatric physician, and confirmed by clinical examination, including routine blood parameters, C-reactive protein, blood sedimentation, electrocardiogram, echocardiography, hematological examination, immunological tests, biochemical examination and imaging signs.

The treatment methods for KD patients are as follows: For patients without CAL, we give a single infusion of IVIG 2 g/kg, and aspirin 30-50 mg/kg/d, orally three times per day, until fever subsided for 3 days. Then, the dose of aspirin was reduced to 3-5 mg/kg per day, once a day, for 6–8 weeks. If the patient was accompanied by CAL, we will continue to give aspirin until the coronary artery returns to normal. Among the 44 KD patients, 1 patient was IVIG non-sensitive KD, and required a second dose of IVIG at 2 g/kg due to persistent or recurrent fever, then received methylprednisolone at 2 mg/kg per day for 3 days, until fever subsided the methylprednisolone was switched to oral and gradually tapered (total course of two weeks), the aspirin was reduced to a maintenance dose for eight weeks.

All the individuals were Han Chinese originating from Ningbo city in Eastern China. Whole blood genomic DNA was extracted by PerkinElmer automatic nucleic acid extractor according to the manuals. DNA concentration was determined by Thermo Scientific™ Nanodrop 2000 platform.

### Bisulfite sequencing

Sequencing primers of NLRC4 were designed by using PyroMark Assay Design 2.0 software. The sequences were 5’-TGGGTAAGGTTATATAGGGTAAATAGA-3’ for the forward primer, 5’- Biotin-ATACAAAAAAATTTCTTCAATCCTCAACT-3’ for the reverse primer, and 5’-TATATAGGGTAAATAGATTATTAGT-3’ for the sequencing primer. All primers were synthesized by Sangon Biotechnology (Sangon, Shanghai, China). 200–500 ng of genomic DNA were bisulfite treated using the EZ DNA Methylation Gold ™ kit (Zymo Research, CA, USA) following the operation manual. Genes were amplified using Zymo Taq Premix (Zymo Research, CA, USA) in a total volume of 20 µL, incluing 10 µl Zymo Taq Premix, 6.5 µl DNase/ RNase-free water, 1 µl primer pairs (20 pM), and 2.5 µL bisulfite-converted DNA. Then sequencing was performed by loading PCR product (10 µl, about 250 ng template) and 3 µl magnetic beads into PyroMark Q48 Autoprep. Finally, the methylation status of each CpG site was analyzed using the Pyro Q CpG software.

### Statistical analyses

Statistical analysis was conducted using SPSS 16.0 (SPSS, Inc., Chicago, IL, USA) and GraphPad Prism 5.0 (GraphPad Software, Inc.). All data are expressed as the Means ± standard error (SEM), *p* < 0.05 was considered statistically significant. Gene methylation levels were analyzed using Student T test or paired sample T test. A more conservative non-parametric approach was used for data which were unable to be normalized. A Pearson regression test was applied for analyzing the correlation of methylation level among five CpG sites, as well as the correlation between methylation level and blood biochemical indexes of KD patients, the Bonferroni correction was applied to adjust the *p*-values. An analysis of covariance (ANCOVA) were performed to compare methylation levels between KD patients and healthy controls using age as a covariate. Receiver operating characteristic (ROC) curves were used to describe and compare the performance of NLRC4 methylation algorithms in diagnostic. The area under the curve and diagnostic performance metrics were calculated from a logistic regression model adjusting for age.

## Results

### Global DNA methylation

**Fig. 1:**
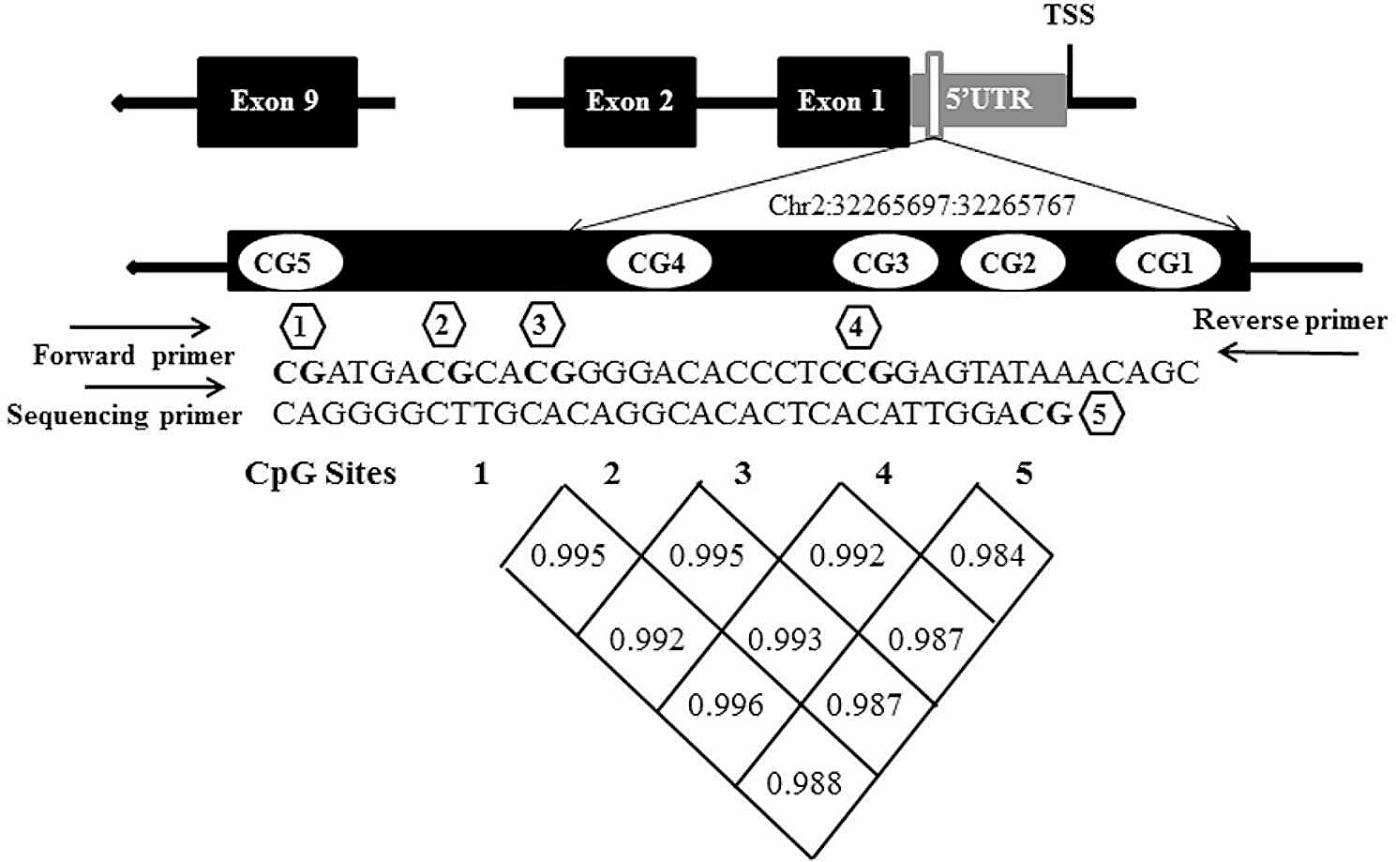
Positions and correlation among 5 CpG sites in NLRC4 promoter. The values in the grid represent the correlation coefficients between CpG sites

As shown in the Fig. 1, five CpG sites were included to represent the methylation of NDRG4 gene. Bisulfite pyrosequencing assay was performed to detected methylation percentages of these five CpG sites in a 70 bp fragment (chr 2:32265697–32,265,767) located in the 5’UTR region (Fig. 1).

**Fig. 2:**
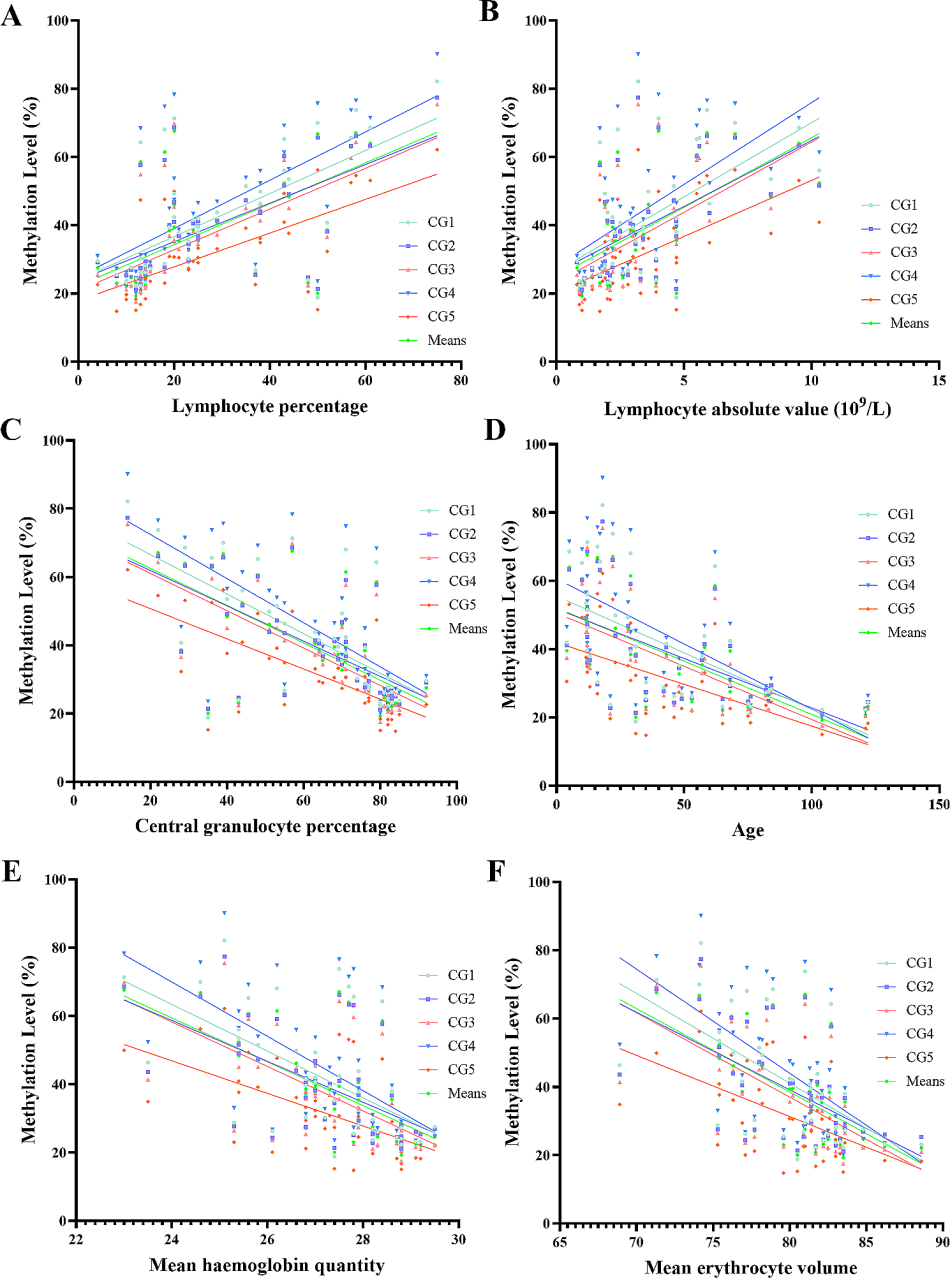
Correlation between NLRC4 methylation and lymphocyte percentagee (**A**), lymphocyte absolute value (**B**), central granulocyte percentage (**C**), age (**D**), mean haemoglobin quantity (**E**) and mean erythrocyte volume (**F**)

Correlation and regression test were conducted to determine the NLRC4 methylation and the associated clinical parameters, in total, 72 quantifiable blood biochemical parameters were detected. In total, NLRC4 methylation are revealed to be associated with 6 parameters, NLRC4 promoter methylation was shown to be significantly negatively correlated with the levels of central granulocyte percentage, age, mean haemoglobin quantity and mean erythrocyte volume. Besides, NLRC4 promoter methylation was positively correlated with lymphocyte percentage, lymphocyte absolute value (Table 1; Fig. 2).

**Table 1:** Correlation analyses between NLRC4 promoter methylation levels and additional variables

Characteristics		Lymphocyte percentage	Lymphocyte absolute value	Central granulocyte percentage	Age	Mean haemoglobin quantity	Mean erythrocyte volume
CG1	Pearson Correlation	0.62237	0.55965	-0.65621	-0.59206	-0.58594	-0.58494
	*p* value	6.47E-06	7.83E-05	1.32E-06	2.30E-05	2.93E-05	3.05E-05
	adjust *p* value (Bonferroni)	0.00299	0.03616	0.00061	0.01064	0.01354	0.01408
CG2	Pearson Correlation	0.63177	0.56302	-0.66514	-0.58552	-0.58246	-0.57328
	*p* value	4.24E-06	6.93E-05	8.43E-07	2.98E-05	3.35E-05	4.75E-05
	adjust *p* value (Bonferroni)	0.00196	0.03203	0.00039	0.01376	0.01550	0.02196
CG3	Pearson Correlation	0.64688	0.58193	-0.68168	-0.60975	-0.61379	-0.60528
	*p* value	2.09E-06	3.42E-05	3.50E-07	1.12E-05	9.39E-06	1.34E-05
	adjust *p* value (Bonferroni)	0.00097	0.01581	0.00016	0.00515	0.00434	0.00621
CG4	Pearson Correlation	0.63001	0.55782	-0.66293	-0.61486	-0.61692	-0.61424
	*p* value	4.60E-06	8.36E-05	9.44E-07	8.97E-06	8.21E-06	9.21E-06
	adjust *p* value (Bonferroni)	0.00212	0.03860	0.00044	0.00414	0.00379	0.00426
CG5	Pearson Correlation	0.66515	0.57648	-0.68360	-0.59511	-0.55840	-0.54520
	*p* value	8.42E-07	4.21E-05	3.15E-07	2.04E-05	8.18E-05	0.000129779
	adjust *p* value (Bonferroni)	0.00039	0.01947	0.00015	0.00942	0.03781	0.05529
Means	Pearson Correlation	0.63977	0.56885	-0.67116	-0.60229	-0.59584	-0.58946
	*p* value	2.93E-06	5.60E-05	6.16E-07	1.52E-05	1.98E-05	2.55E-05
	adjust *p* value (Bonferroni)	0.00135	0.02588	0.00028	0.00703	0.00914	0.01180

**Fig. 3:**
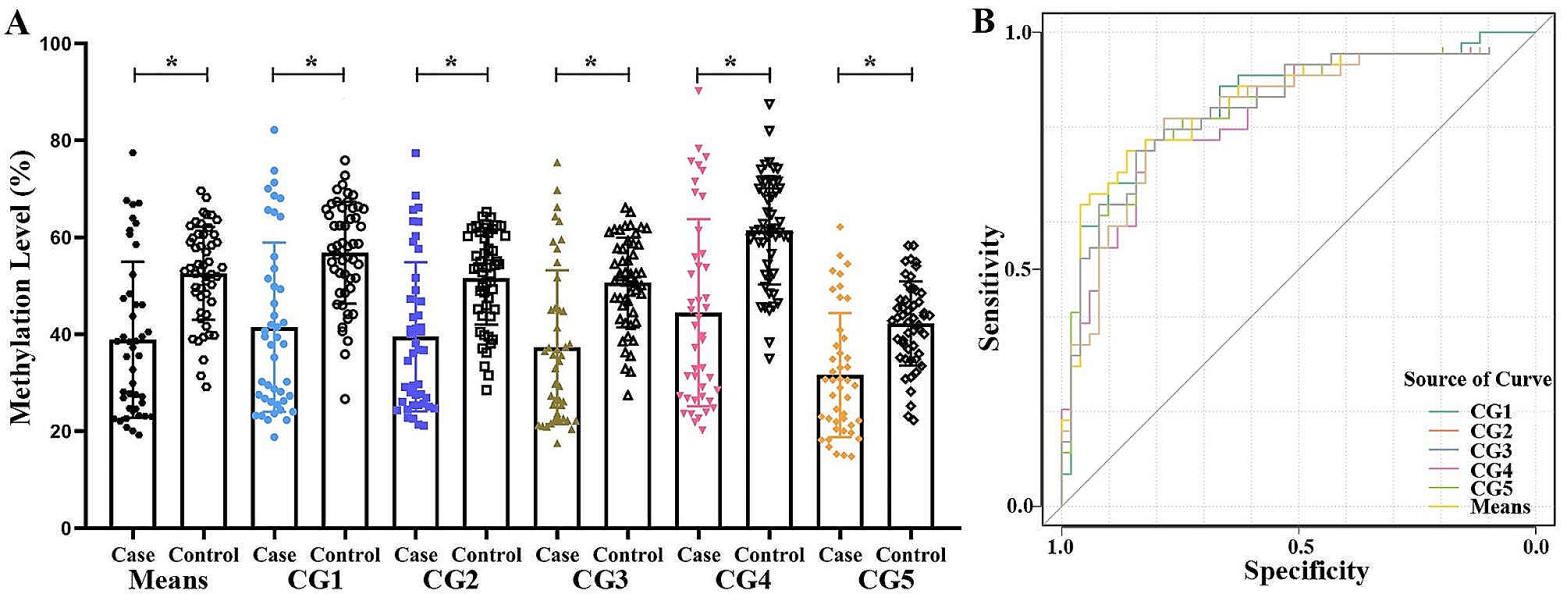
NLRC4 methylation levels and diagnostic possibility. (**A**) Comparison of NLRC4 methylation levels between Kawasaki disease (KD) patients and controls; (**B**) Adjusted generalized ROC curve. A subject is assessed as disease (positive) or healthy (negative) according to the ROC curve. *p < 0.0001, ROC: Receiver operating characteristic

Significant correlations among five CpG sites in NLRC4 were found (*r* > 0.98, *p* < 0.05, Fig. 1), so the average value for these five CpG sites were also used to analyze the overall methylation situation. Two-tailed T-test demonstrated that the mean methylation level of NLRC4 in KD was significantly lower than that in the control group (KD: 38.87 ± 2.43, Control: 52.57 ± 1.35, *p* = 5.42E-06, Table 2; Fig. 3A).

A further subgroup analysis showed that the methylation levels of the NLRC4 gene differed significantly between KD patients and controls in the age groups of less than 36 months and more than 36 months (*p* < 0.001 for both groups, Table 2). Furthermore, significant differences in NLRC4 methylation were detected only in males, but not in females (Table 2). The gender difference might be due to the few female KD patients were enrolled.

**Table 2:** Comparisons of NLRC4 methylation levels between cases and controls

Terms	Characteristics	Case	Control	*p* value^*^
**All**	All	44	51	
	Month Age	42.28 ± 4.68	51.35 ± 2.02	0.081
	CpG1(%)	41.46 ± 2.63	56.84 ± 1.48	**2.93E-06**
	CpG2(%)	39.49 ± 2.32	51.64 ± 1.35	**2.35E-05**
	CpG3(%)	37.36 ± 2.39	50.68 ± 1.30	**6.46E-06**
	CpG4(%)	44.45 ± 2.91	61.45 ± 1.56	**2.47E-06**
	CpG5(%)	31.58 ± 1.93	42.24 ± 1.22	**1.26E-05**
	Means methylation(%)	38.87 ± 2.43	52.57 ± 1.35	**5.42E-06**
**Age**	≤ 36 months	24	10	
	CpG1(%)	49.98 ± 3.62	65.42 ± 1.93	**0.00069**
	CpG2(%)	46.86 ± 3.24	59.85 ± 1.55	**0.00107**
	CpG3(%)	45.42 ± 3.29	58.358 ± 1.80	**0.00163**
	CpG4(%)	54.31 ± 3.87	70.749 ± 2.646	**0.00137**
	CpG5(%)	37.62 ± 2.71	49.89 ± 1.938	**0.00087**
	Means methylation(%)	46.84 ± 3.33	60.85 ± 1.88	**0.00091**
	> 36 months	20	41	
	CpG1(%)	31.24 ± 2.32	54.75 ± 1.62	**7.98E-15**
	CpG2(%)	30.65 ± 1.99	49.63 ± 1.48	**8.98E-13**
	CpG3(%)	27.68 ± 1.93	48.81 ± 1.408	**1.13E-15**
	CpG4(%)	32.62 ± 2.71	59.186 ± 1.653	**1.62E-16**
	CpG5(%)	24.32 ± 1.65	40.373 ± 1.28	**2.02E-12**
	Means methylation(%)	29.30 ± 2.09	50.55 ± 1.45	**5.49E-15**
**Gender**	Male	31	46	
	CpG1(%)	43.98 ± 3.49	58.26 ± 1.38	**4.87E-04**
	CpG2(%)	41.62 ± 3.09	52.77 ± 1.33	**0.002**
	CpG3(%)	39.89 ± 3.14	51.85 ± 1.25	**0.001**
	CpG4(%)	47.55 ± 3.80	62.75 ± 1.54	**6.40E-04**
	CpG5(%)	33.21 ± 2.57	43.18 ± 1.20	**0.001**
	Means methylation(%)	41.25 ± 3.21	53.76 ± 1.30	**8.38E-04**
	Female	13	5	
	CpG1(%)	35.47 ± 2.66	43.85 ± 5.77	0.15
	CpG2(%)	34.43 ± 2.31	41.20 ± 4.28	0.156
	CpG3(%)	31.32 ± 2.48	39.92 ± 4.57	0.098
	CpG4(%)	37.05 ± 3.14	49.49 ± 4.94	0.052
	CpG5(%)	27.67 ± 1.95	33.54 ± 4.37	0.172
	Means methylation(%)	33.19 ± 2.49	41.60 ± 4.71	0.108

* The *p* value less than or equal to 0.05 is in bold

Considering the significant correlation between age and methylation levels. We first tested the homogeneity of regression slopes assumption by including an interaction term between group and age in the model. The interaction term was not statistically significant (*p* > 0.05), indicating the regressions lines were parallel and the assumption was met. We then performed ANCOVA with age as covariate, there were still statistically significant differences in methylation levels between KD patients and control groups (all *p*-values ≤ 1.74E-08, Table 3). In summary, even when accounting for age effects, methylation levels differed significantly between KD patients and controls.

**Table 3:** Analysis of covariance of methylation levels between Kawasaki disease cases and healthy controls with age as a covariate

Positions	Estimated value(%)				Mean square	F	*p* value
	Case		Control				
	Means	SEM	Means	SEM			
CG1	40.069^a^	1.905	58.047^a^	1.767	7358.594	47.004	**7.95E-10**
CG2	38.258^a^	1.699	52.705^a^	1.576	4752.4	38.17	**1.74E-08**
CG3	36.070^a^	1.699	51.798^a^	1.576	5632.186	45.248	**1.45E-09**
CG4	42.852^a^	2.044	62.833^a^	1.896	9090.251	50.429	**2.54E-10**
CG5	30.531^a^	1.454	43.142^a^	1.349	3621.201	39.715	**1.00E-08**
Means	37.556^a^	1.738	53.705^a^	1.612	5937.843	45.554	**1.30E-09**

a: The covariates appearing in the model are valued according to the following values Age = 47.1526

ROC curve was used to evaluate the diagnostic ability of NLRC4 gene methylation for KD. Statistical analysis showed that the five CpG sites and mean methylation level of NLRC4 were statistically significant for the classifiers in KD (*p* ≤ 4.11E-05), and the areas under ROC curve were all greater than 0.82 (Fig. 3B), and the sensitivity were all greater than 0.75 as shown in Fig. 3B; Table 4. Such as, the predictive probability of mean NLRC4 methylation level to discriminate KD patients vs. controls based on ROC curve (95% CI) was 0.844 (0.762–0.926). The cutoff (48.56%) exhibited an 75% sensitivity rate and a 84% specificity rate for predicting KD. Additional analyses using different subsets of CG sites as predictors have also been performed. A total of 25 combinations including 2 loci, 3 loci, and 4 loci was conducted, all of these combinations had AUC values greater than 0.83 (Supplementary Table S1).

**Fig. 4:**
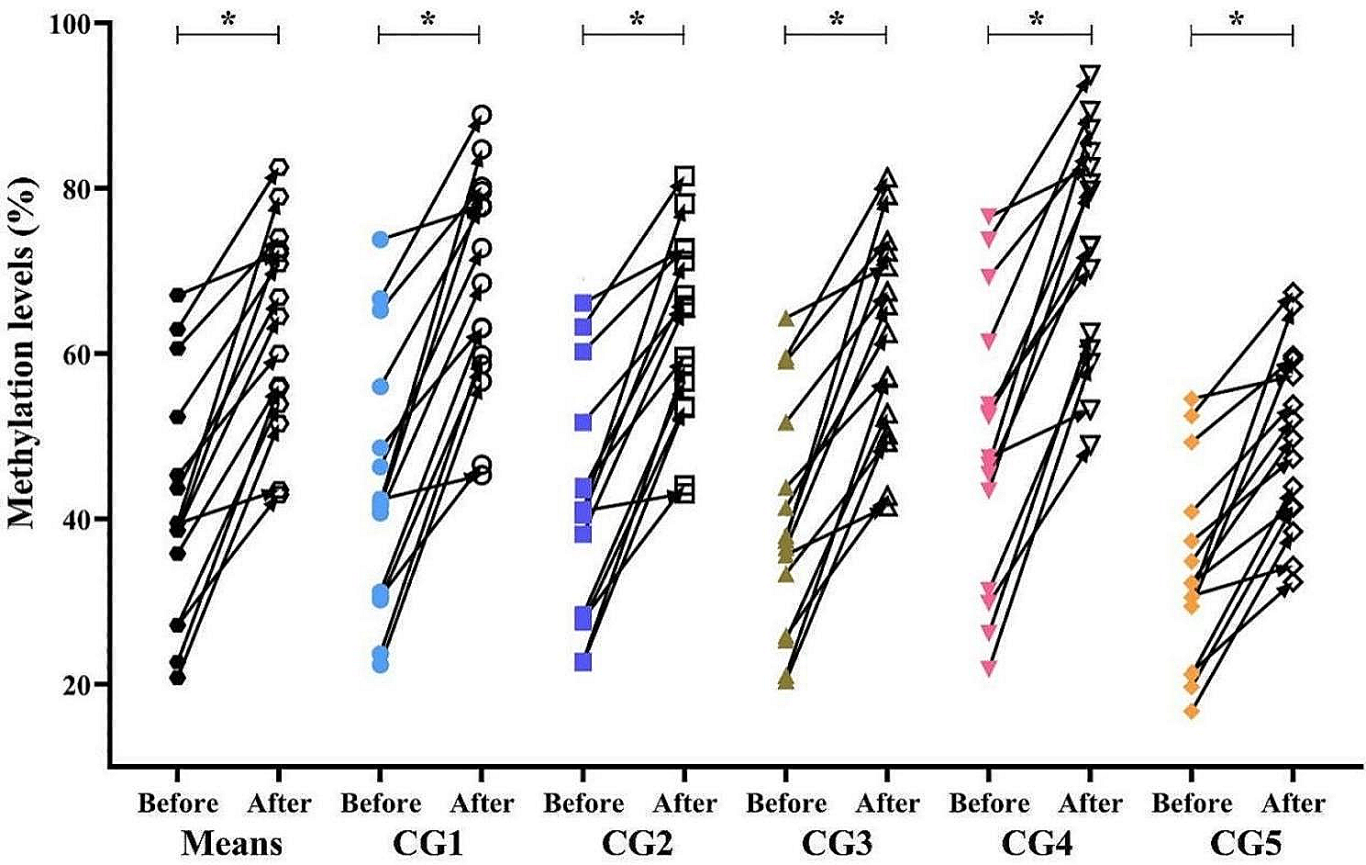
Methylation levels of NLRC4 before and after IVIG treatment in 15 Kawasaki disease patients. IVIG: intravenous immunoglobulin, *p < 0.0001

Subsequently, the methylation changes of NLRC4 were evaluated before and after IVIG treatment. The results demonstrated that mean methylation levels of five CpG sites increased significantly after IVIG treatment (CG1: 44.02 ± 4.03 vs. 68.25 ± 3.46, *p* = 1.69E-06; CG2: 41.26 ± 3.66 vs. 62.92 ± 2.98; *p* = 1.32E-05; CG3: 39.60 ± 3.61 vs. 61.61 ± 3.27, *p* = 1.59E-06; CG4: 48.79 ± 4.35 vs. 73.18 ± 3.56, *p* = 5.80E-07; CG5: 33.58 ± 3.03 vs. 49.64 ± 2.83, *p* = 8.50E-06, Fig. 4). The overall methylation level of NLRC4 remarkably increase after IVIG treatment (41.45 ± 3.70 vs. 63.12 ± 3.18, *p* = 1.11E-06, Fig. 4).

**Table 4:** The predictive probability to discriminate Kawasaki Disease patients vs. controls based on Receiver operating characteristic curve adjusting for age

Test Result Variable(s)	Cut Off	Sensitivity	Specificity	*P* value	AUC	Asymptotic 95% Confidence Interval	
						Lower Bound	Upper Bound
Means	48.56	0.75	0.84	9.61E-06	0.844	0.762	0.926
CG1	39.70	0.81	0.78	1.08E-05	0.855	0.776	0.934
CG2	44.51	0.77	0.82	4.11E-05	0.828	0.744	0.912
CG3	46.31	0.77	0.82	9.44E-06	0.844	0.763	0.925
CG4	53.10	0.75	0.86	9.60E-06	0.856	0.777	0.935
CG5	39.98	0.81	0.78	1.52E-05	0.835	0.751	0.918

## Discussion

In the current study, we investigated the NLRC4 methylation changes in KD patients. The NLRC4 methylation in the KD patients significantly reduced compared to normal control. And ROC curve showed that methylation at each site of the NLRC4 gene, as well as at combinations of sites could serve as potential adjunctive diagnostic biomarkers and exhibited high specificity and sensitivity, which extends the diagnosis beyond the major findings in the acute phase. Further analysis showed that the methylation levels increased after IVIG treatment. There was an age-inverse dependent pattern in the induced methylation levels of NLRC4 promoter in the KD patients. And NLRC4 methylation level was significantly correlated with 6 clinical parameters, including lymphocyte percentagee, lymphocyte absolute value, central granulocyte percentage, age, mean haemoglobin quantity and mean erythrocyte volume.

As a member of the protein family of cytoplasmic immune receptors, NLRC4 is expressed in immune cells such as monocytes, macrophages, and neutrophils [13]. The NLRC4 gene encodes a pattern recognition receptor involved in inflammasome formation that can recognize bacterial flagellin and rod proteins, thereby activating inflammatory factors. The activation of NLRC4 inflammasome and mutations in NLRC4 are linked to autoinflammatory disorders in humans [14, 15]. And DNA methylation is an important epigenetic mechanism regulating gene expression. Our results are consistent with one previous study, which explained that epigenetics hypomethylation and up-regulation of NLRC4 in KD disease [12]. This is also verified that DNA hypomethylation in promoter plays a critical role in activing NLRC4 gene. Previous study showed that activation of the NLRC4 inflammasome leads to maturation of caspase-1, then mediates the excessive secretion of proinflammatory cytokines such as interleukin-1b (IL-1b), IL-1β and IL-18 [16, 17]. Strikingly, activation of the NLRC4 inflammasome also promoted expression of the costimulatory receptor CD80 as well as expression of immunoregulatory receptors PD-L1 and Siglec-8 [18]. This likely represents a major pathway by which reduced NLRC4 methylation promotes systemic inflammation in KD. Additionally, NLRC4 collaborates with the NLRP3 inflammasome to mediate protective immunity against protozoan parasite infection, as evidenced by impaired cytokine production and increased pathogen susceptibility in NLRC4/NLRP3 double knockout cells [19], so its hypomethylation may impair this synergy. Barrier disruption can also activate the FPR1-NLRC4 axis and exacerbate inflammation [20]. Thus, NLRC4 hypomethylation may affect its expression and function, then may promote KD inflammation through multiple interconnected mechanisms involving inflammasome activation, inflammatory cytokine release, and disruption of NLRP3 cross-talk. NLRC4 hypomethylation and overexpression are likely critical drivers of KD. In skin barrier disruption models, Nlrc4 silencing can mitigate inflammation [20], suggesting NLRC4 inflammasome inhibition may be a potential therapeutic target for alleviating inflammation in KD.

And many previous studies showed that there exist significant differences in methylation levels of genes involved in the innate immune system, such as TLR1, 2, 4, 6, 8 and 9 genes in KD patients and normal controls. These genes can induce the inflammatory cytokines and may mediate the immune pathogenesis of KD [1]. Chang Kuo et al. [21] found that the mRNA expression levels of matrix metalloproteinase MMP-8, -9 and − 25 in KD patients were significantly up-regulated compared with healthy controls, genes methylation were directly involved in this change, and these changes were reversed after IVIG treatments. Moreover, Danqi Chang et al. [22] confirmed that MAPK14 and PHLPP1 genes are hypomethylation and high expression in KD disease. Understanding the roles of these genetic alterations regulated by gene methylation has implications for potential therapeutic approaches. Further studies based on these findings will help establish new treatment strategies in the future.

High-dose IVIG is a widely used therapeutic approach for KD. IVIG contains anti-idiotypes against autoantibodies and may be effective in the treatment of some autoimmune diseases through idiotypic/anti-idiotypic interactions [23]. Specifically, IVIG has complex immunomodulatory effects on cytokine production, immune cell activation, and gene expression. Existing methylation microarray studies have shown that CpG sites are mostly hypermethylated after IVIG treatment [24]. Extensive methylation changes may be an important mechanism by which IVIG exerts immune regulation. And genes near hypermethylated CpG sites are enriched in pathways associated with inflammatory immune responses, such as hematopoietic cell differentiation, cytokine-cytokine receptor interaction, chemokine signaling pathway, and Jak-STAT signaling pathway [24]. In the present study, methylation levels of NLRC4 significantly increased after IVIG treatment, which could be an explanation for previous study that once KD patients underwent IVIG treatment, mRNA levels of NLRC4 considerably decreased [12], and it may reflect part of the extensive epigenetic changes induced by IVIG treatment. Previous studies have made similar findings, such as the significant correlation between low-affinity immunoglobulin Fc receptor II-a (FCGR2A) promoter methylation, KD susceptibility, and IVIG treatment efficacy [25]. On the other hand, IVIG treatment can enhance the immunosuppressive function of regulatory T cells, and methylation changes in Treg related genes play an important regulatory role in this process [26]. In addition, increased FcγRIIA expression is associated with KD susceptibility. In KD patients receiving IVIG treatment, methylation of Fcγ receptors was significantly increased, and their expression was related to IVIG efficacy and vascular lesions [27]. In summary, the increase in NLRC4 methylation may be a manifestation of the multiple immunomodulatory effects of IVIG, and its mechanism may be related to the influence of IVIG on DNA methyltransferases, inflammatory gene expression, Treg function, and other aspects. These existing evidences can be valuable supplements and support for our preliminary findings, but further functional studies are needed for validation.

Correlation analyses were also performed between NLRC4 promoter methylation level and 73 clinical quantifiable indicators. Among them, 6 significant associations were observed. The positive correlation between NLRC4 methylation levels and lymphocyte percentages may imply that low methylation level of the NLRC4 gene leads to its high expression, thereby enhancing the activation and proliferation of lymphocytes, thus participating in the immunopathological process of KD. The negative correlation with neutrophil percentages indicates that low expression of NLRC4 may inhibit neutrophil function. Multicenter studies had proved that Neutrophil: Lymphocyte Ratio might work as an independent predictor of coronary artery lesions and IVIG resistance in KD [28, 29]. Further study is needed to elucidate the molecular mechanisms. This finding preliminarily reveals the potential role of NLRC4 gene methylation in immune and inflammatory responses in KD, which helps us further elucidate the pathogenesis of KD. However, functional validation is still needed to confirm its role and mechanisms.

There are several limitations of our study that need to be taken into consideration. Firstly, only 5 CpG sites were detected which might not a comprehensive representation of the overall methylation level of NLRC4 methylation to KD patients. Secondly, we did not examine the interaction of methylation levels of the NLRC4 gene with other relevant inflammatory cytokines. Thirdly, there were only 13 female cases and 5 female healthy controls were enrolled in our study. The small sample size of 44 KD patients is a limitation of this study. Further research with larger cohorts is warranted to validate the findings. Fourthly, we did not compare the IVIG responders and non-responders, which may have different clinical characteristics and outcomes. Lastly, in this study, we only explored the regulation mechanism by which IVIG inhibits excessive inflammation. However, we cannot rule out the role of aspirin, the main ingredient of which was salicylic acid, and aspirin is often administered with IVIG to alleviate excessive inflammatory immune response. Therefore, the effect of salicylic acid on methylation alteration also deserves more attention.

## Conclusions

Our study suggested that there was a significant contribution of NLRC4 promoter hypomethylation to the risk of KD. Aberrant NLRC4 methylation in peripheral blood is associated with IVIG treatment response. The significant correlation with multiple clinical parameters suggests NLRC4 methylation may have potential as a predictive biomarker. However, further research is needed to determine whether it may have potential as a therapeutic target. Subsequent prognostic data of patients treated with IVIG should be followed to further confirm these assumptions.

### Electronic supplementary material

Below is the link to the electronic supplementary material.

Supplementary Material 1

Supplementary Material 2

## Data Availability

The datasets used in the current study are available from the attached file (Appendix 1).

## Abbreviations

KD: Kawasaki disease
NLRC4: NLR Family CARD Domain Containing 4
IVIG: Intravenous immunoglobulin
ROC: Receiver operating characteristic
CAA: Coronary artery aneurysm
MAS: Macrophage activation syndrome

## References

[1] Huang YH, Li SC, Huang LH, Chen PC, Lin YY, Lin CC (2017). Identifying genetic hypomethylation and upregulation of toll-like receptors in Kawasaki disease. Oncotarget.

[2] Newburger JW, Takahashi M, Beiser AS, Burns JC, Bastian J, Chung KJ (1991). A single intravenous infusion of gamma globulin as compared with four infusions in the treatment of acute Kawasaki syndrome. N Engl J Med.

[3] Newburger JW, Takahashi M, Burns JC (2016). Kawasaki Disease. J Am Coll Cardiol.

[4] Tsoukas P, Yeung RSM (2022). Kawasaki disease and MIS-C share a host immune response. Nat Rev Rheumatol.

[5] Fujita Y, Nakamura Y, Sakata K, Hara N, Kobayashi M, Nagai M (1989). Kawasaki disease in families. Pediatrics.

[6] Uehara R, Yashiro M, Nakamura Y, Yanagawa H (2003). Kawasaki disease in parents and children. Acta Paediatr.

[7] Shulman ST, Rowley AH (2015). Kawasaki disease: insights into pathogenesis and approaches to treatment. Nat Rev Rheumatol.

[8] Ikeda K, Yamaguchi K, Tanaka T, Mizuno Y, Hijikata A, Ohara O (2010). Unique activation status of peripheral blood mononuclear cells at acute phase of Kawasaki disease. Clin Exp Immunol.

[9] Li T, Ortiz-Fernandez L, Andres-Leon E, Ciudad L, Javierre BM, Lopez-Isac E (2020). Epigenomics and transcriptomics of systemic sclerosis CD4 + T cells reveal long-range dysregulation of key inflammatory pathways mediated by disease-associated susceptibility loci. Genome Med.

[10] Shahi A, Afzali S, Firoozi Z, Mohaghegh P, Moravej A, Hosseinipour A (2023). Potential roles of NLRP3 inflammasome in the pathogenesis of Kawasaki disease. J Cell Physiol.

[11] Guo H, Callaway JB, Ting JP (2015). Inflammasomes: mechanism of action, role in disease, and therapeutics. Nat Med.

[12] Huang YH, Lo MH, Cai XY, Kuo HC (2018). Epigenetic hypomethylation and upregulation of NLRC4 and NLRP12 in Kawasaki disease. Oncotarget.

[13] Raghawan AK, Ramaswamy R, Radha V, Swarup G (2019). HSC70 regulates cold-induced caspase-1 hyperactivation by an autoinflammation-causing mutant of cytoplasmic immune receptor NLRC4. Proc Natl Acad Sci U S A.

[14] Matico RE, Yu X, Miller R, Somani S, Ricketts MD, Kumar N et al. Structural basis of the human NAIP/NLRC4 inflammasome assembly and pathogen sensing. Nat Struct Mol Biol. 2024. 10.1038/s41594-023-01143-z PMC10803261 38177670

[15] Sundaram B, Kanneganti TD. Advances in understanding activation and function of the NLRC4 inflammasome. Int J Mol Sci. 2021;22. 10.3390/ijms22031048 PMC7864484 33494299

[16] Eeckhout E, Asaoka T, Van Gorp H, Demon D, Girard-Guyonvarc’h C, Andries V (2023). The autoinflammation-associated NLRC4(V341A) mutation increases microbiota-independent IL-18 production but does not recapitulate human autoinflammatory symptoms in mice. Front Immunol.

[17] Javaid N, Hirai H, Che FS, Choi S. Molecular basis for the activation of human innate Immune response by the Flagellin Derived from Plant-pathogenic bacterium, Acidovorax avenae. Int J Mol Sci. 2021;22. 10.3390/ijms22136920 PMC8268093 34203170

[18] Akkaya I, Oylumlu E, Ozel I, Uzel G, Durmus L, Ciraci C (2021). NLRC4 inflammasome-mediated regulation of eosinophilic functions. Immune Netw.

[19] Amaral MP, Cardoso FD, de Farias IS, de Souza RQ, Matteucci KC, Torrecilhas AC (2023). NAIP/NLRC4 inflammasome participates in macrophage responses to Trypanosoma Cruzi by a mechanism that relies on cathepsin-dependent caspase-1 cleavage. Front Immunol.

[20] Shao S, Sun Z, Chu M, Chen J, Cao T, Swindell WR et al. FPR1 contributes to epidermal barrier dysfunction-induced skin inflammation through NLRC4-dependent keratinocyte activation. Br J Dermatol. 2023. 10.1093/bjd/ljad455 37979162

[21] Kuo HC, Li SC, Huang LH, Huang YH (2017). Epigenetic hypomethylation and upregulation of matrix metalloproteinase 9 in Kawasaki disease. Oncotarget.

[22] Chang D, Qian C, Li H, Feng H (2019). Comprehensive analyses of DNA methylation and gene expression profiles of Kawasaki disease. J Cell Biochem.

[23] Sultan Y, Kazatchkine MD, Maisonneuve P, Nydegger UE (1984). Anti-idiotypic suppression of autoantibodies to factor VIII (antihaemophilic factor) by high-dose intravenous gammaglobulin. Lancet.

[24] Li SC, Chan WC, Huang YH, Guo MM, Yu HR, Huang FC (2016). Major methylation alterations on the CpG markers of inflammatory immune associated genes after IVIG treatment in Kawasaki disease. BMC Med Genomics.

[25] Kuo HC, Hsu YW, Wu MS, Woon PY, Wong HS, Tsai LJ et al. FCGR2A Promoter Methylation and Risks for Intravenous Immunoglobulin Treatment Responses in Kawasaki Disease. Mediators Inflamm. 2015;2015:564625. 10.1155/2015/564625 PMC4451985 26089602

[26] Piotrowska M, Gliwinski M, Trzonkowski P, Iwaszkiewicz-Grzes D. Regulatory T cells-related genes are under DNA methylation influence. Int J Mol Sci. 2021; 22. 10.3390/ijms22137144 PMC8267835 34281195

[27] Chang LS, Lo MH, Li SC, Yang MY, Hsieh KS, Kuo HC (2017). The effect of FcgammaRIIA and FcgammaRIIB on coronary artery lesion formation and intravenous immunoglobulin treatment responses in children with Kawasaki disease. Oncotarget.

[28] Chidambaram AC, Ramamoorthy JG, Anantharaj A (2023). Neutrophil-lymphocyte ratio for Predicting Coronary Artery lesions in Children with Kawasaki Disease. Indian Pediatr.

[29] Lu Y, Tang Y, Wang B, Li X, Xu Q, Chu H (2022). Predicting immunoglobulin resistance in Kawasaki disease: an assessment of neutrophil to lymphocyte platelet ratio. Ital J Pediatr.

