# Supplementary material for: NLRC4 methylation and its response to intravenous immunoglobulin therapy in Kawasaki disease: a case control study
Source: BMC Pediatr. 2024 Mar 16;24:190. doi: 10.1186/s12887-024-04672-8 (PMC10943762; doi:10.1186/s12887-024-04672-8)
Supplement: Supplementary file 2 — Supplementary Material 2 [file 12887_2024_4672_MOESM2_ESM.docx]

Table S1: The predictive probability to discriminate Kawasaki Disease patients vs controls based on Receiver operator curve

for different combinations of CG sites as predictors

| **Test Result  Variable(s)** | **Sensitivity** | **Specificity** | **P value** | **AUC** | **Asymptotic 95%  Confidence Interval** | |
| --- | --- | --- | --- | --- | --- | --- |
|  |  |  |  |  | **Lower  Bound** | **Upper  Bound** |
| Mean (CG1+2) | 0.773 | 0.824 | 1.45E-05 | 0.8409 | 0.7588 | 0.9230 |
| Mean (CG1+3) | 0.795 | 0.804 | 7.94E-06 | 0.8507 | 0.7712 | 0.9302 |
| Mean (CG1+4) | 0.659 | 0.941 | 9.27E-06 | 0.8538 | 0.7735 | 0.9342 |
| Mean (CG1+5) | 0.795 | 0.804 | 1.03E-05 | 0.8467 | 0.7657 | 0.9277 |
| Mean (CG2+3) | 0.795 | 0.804 | 1.47E-05 | 0.8360 | 0.7532 | 0.9188 |
| Mean (CG2+4) | 0.773 | 0.843 | 1.47E-05 | 0.8449 | 0.7633 | 0.9265 |
| Mean (CG2+5) | 0.750 | 0.843 | 1.93E-05 | 0.8329 | 0.7492 | 0.9165 |
| Mean (CG3+4) | 0.636 | 0.961 | 9.28E-06 | 0.8507 | 0.7704 | 0.9310 |
| Mean (CG3+5) | 0.773 | 0.804 | 1.05E-05 | 0.8405 | 0.7589 | 0.9220 |
| Mean (CG4+5) | 0.773 | 0.824 | 8.22E-06 | 0.8498 | 0.7691 | 0.9305 |
| Mean (CG1+2+3) | 0.773 | 0.824 | 1.20E-05 | 0.8422 | 0.7603 | 0.9242 |
| Mean (CG1+2+4) | 0.773 | 0.843 | 1.24E-05 | 0.8489 | 0.7679 | 0.9300 |
| Mean (CG1+2+5) | 0.750 | 0.843 | 1.20E-05 | 0.8400 | 0.7578 | 0.9222 |
| Mean (CG1+3+4) | 0.636 | 0.961 | 8.07E-06 | 0.8525 | 0.7722 | 0.9328 |
| Mean (CG1+3+5) | 0.750 | 0.843 | 8.81E-06 | 0.8480 | 0.7682 | 0.9279 |
| Mean (CG1+4+5) | 0.773 | 0.824 | 8.66E-06 | 0.8516 | 0.7716 | 0.9316 |
| Mean (CG2+3+4) | 0.773 | 0.824 | 1.05E-05 | 0.8458 | 0.7646 | 0.9270 |
| Mean (CG2+3+5) | 0.795 | 0.784 | 1.52E-05 | 0.8360 | 0.7530 | 0.9190 |
| Mean (CG2+4+5) | 0.773 | 0.824 | 1.20E-05 | 0.8445 | 0.7628 | 0.9261 |
| Mean (CG3+4+5) | 0.773 | 0.824 | 9.44E-06 | 0.8471 | 0.7665 | 0.9278 |
| Mean (CG1+2+3+4) | 0.750 | 0.843 | 9.28E-06 | 0.8453 | 0.7638 | 0.9270 |
| Mean (CG1+2+3+5) | 0.796 | 0.784 | 1.05E-05 | 0.8405 | 0.7584 | 0.9225 |
| Mean (CG1+2+4+5) | 0.773 | 0.824 | 1.05E-05 | 0.8458 | 0.7642 | 0.9273 |
| Mean (CG1+3+4+5) | 0.773 | 0.824 | 8.22E-06 | 0.8476 | 0.7668 | 0.9283 |
| Mean (CG2+3+4+5) | 0.750 | 0.843 | 1.16E-05 | 0.8427 | 0.7608 | 0.9246 |
